# A Symmetrical Leech-Inspired Soft Crawling Robot Based on Gesture Control

**DOI:** 10.3390/biomimetics10010035

**Published:** 2025-01-08

**Authors:** Jiabiao Li, Ruiheng Liu, Tianyu Zhang, Jianbin Liu

**Affiliations:** 1 Key Laboratory of Mechanism Theory and Equipment Design, Ministry of Education, Tianjin University, Tianjin 300072, China; lijiabiao26@tju.edu.cn (J.L.); ruihengliu@zju.edu.cn (R.L.); zhang_tianyu@tju.edu.cn (T.Z.); 2 School of Mechanical Engineering, Zhejiang University, Hangzhou 310058, China

**Keywords:** Leap Motion, gesture recognition, TPU, soft robot, BP neural network

## Abstract

This paper presents a novel soft crawling robot controlled by gesture recognition, aimed at enhancing the operability and adaptability of soft robots through natural human–computer interactions. The Leap Motion sensor is employed to capture hand gesture data, and Unreal Engine is used for gesture recognition. Using the UE4Duino, gesture semantics are transmitted to an Arduino control system, enabling direct control over the robot’s movements. For accurate and real-time gesture recognition, we propose a threshold-based method for static gestures and a backpropagation (BP) neural network model for dynamic gestures. In terms of design, the robot utilizes cost-effective thermoplastic polyurethane (TPU) film as the primary pneumatic actuator material. Through a positive and negative pressure switching circuit, the robot’s actuators achieve controllable extension and contraction, allowing for basic movements such as linear motion and directional changes. Experimental results demonstrate that the robot can successfully perform diverse motions under gesture control, highlighting the potential of gesture-based interaction in soft robotics.

## 1. Introduction

In recent years, with the rapid advancement of robotics technology, soft robots have shown great potential in fields such as industry, healthcare, and rescue operations due to their excellent flexibility, infinite degrees of freedom, and adaptability to complex environments [1,2,3,4,5]. Unlike traditional rigid robots, which are limited in their movements by fixed structures, soft robots can perform highly flexible motions through deformation, making them well suited for navigating irregular surfaces and obstacles [6,7]. However, controlling and driving these soft robots pose significant challenges. Traditional control methods, such as programmable logic controllers (PLCs), manual switches, and sensor-based feedback systems, are typically effective for simple and repetitive tasks but often lack the flexibility and real-time response required for more dynamic and complex applications. The common driving methods for soft robots include pneumatic [8,9,10], electric [11,12], and chemical [13,14,15]. However, these methods have certain drawbacks. For instance, achieving precise control in multi-degree-of-freedom movements can be challenging. Additionally, it is difficult to realize detailed deformation in specific environments. The uncontrollability and irreversibility of chemical reactions also limit the application of robots in complex and repetitive tasks.

The maturity of gesture recognition technology in recent years provides a new approach for interaction in robot control [16]. By capturing and interpreting human gestures, operators can directly control robots in a natural and intuitive way, offering a higher level of flexibility that is particularly suitable for high-degree-of-freedom soft robots. Gesture-based control eliminates the need for complex physical interfaces, allowing the operator to initiate different motions with a simple hand movement, thereby enhancing both the ease of use and adaptability in multi-mode control. While various gesture-based control techniques have been widely applied in robotic arms, drones, and other devices, their application in soft robotics remains in the exploratory stage, especially regarding the realization of complex, multi-degree-of-freedom motion control.

The earliest gesture interaction device was a multi-sensor fusion data glove [17], which can recognize simple hand movements, such as pressing and lifting. However, its accuracy was not perfect. With the introduction of Leap Motion, an increasing number of researchers have turned to it for gesture studies. The articulated robotic arm is controlled by Leap Motion [18], and each joint of the arm is equipped with a HITEC servo motor, which is controlled by Pulse Width Modulation (PWM) waves. The angle of rotation of each motor is controlled by the gestures recognized by Leap Motion. Additionally, the delta parallel robot is controlled by the eight types of gestures defined by Leap Motion, ensuring high precision and efficiency [19]. A micro-rotor vehicle control method based on natural interaction behaviors has been studied [20]. Additionally, other maneuvers using gesture-based control can also be remotely operated on the DLR-HIT IIxing4 manipulator. In this system, sensors capture the coordinate positions of the human fingers, and inverse kinematics is employed to convert the Cartesian position data of the fingertips into the corresponding joint angles of all five fingers [21]. This information can then be sent directly to the remote operator through user data protocols. Similarly, a gesture-based control system for a soft rotary actuator has also been designed [22]. This actuator has a range of motion from 0 to 90 degrees for positive pressure actuation and from 0 to −16 degrees for negative pressure actuation. The designed gesture commands include circle, swipe, screen tap, and key tap gestures, which are used to generate clockwise and counterclockwise movements, as well as stop and start motions for the rotary actuator. Danilo Avola utilized the angles formed by human finger bones as a feature in training recurrent neural networks for the Leap Motion gesture recognition algorithm [23]. Recurrent neural networks are well suited for analyzing gesture data since they effectively model the contextual information of time series, achieving a recognition accuracy of over 96%. Chunxu Li researched a Leap Motion sensor-based controller to track the hand movements of the operator and used a Kalman filtering algorithm to process the data signals [24]. Additionally, Hua Li proposed a Spatial Fuzzy Matching (SFM) algorithm that creates a fused gesture data set by matching and integrating spatial information [25]. As a result, the recognition rate for static gestures reaches between 94% and 100%.

In this study, we propose a gesture-controlled soft robotic system that combines the intuitive control provided by Leap Motion sensors with the powerful and flexible actuation of a pneumatic system to enhance its operability in complex environments. The system uses the Leap Motion sensors to capture hand gesture data, with Unreal Engine performing gesture recognition. Recognized gesture signals are then transmitted to an Arduino control system via the UE4Duino, where they are converted into movement commands. These commands directly influence the pneumatic actuators, enabling real-time control of the soft robot’s movements. To ensure accuracy in gesture recognition, this paper employs a threshold-based method for static gestures and a BP neural network model for dynamic gestures, significantly reducing recognition errors. Compared to traditional control methods, the gesture-based control approach proposed in this study combines the natural interactive capabilities of gesture recognition with the powerful and adaptive drive provided by pneumatics, achieving multi-degree-of-freedom, multi-modal control and offering significant improvements in ease of operation. Through this study, we aim to provide new technical support and practical references for the further development of soft robots in multi-degree-of-freedom control and human–machine interaction applications.

## 2. Gesture Design and Recognition Model Establishment

### 2.1. Theory of Gesture Recognition

**Figure 1:**
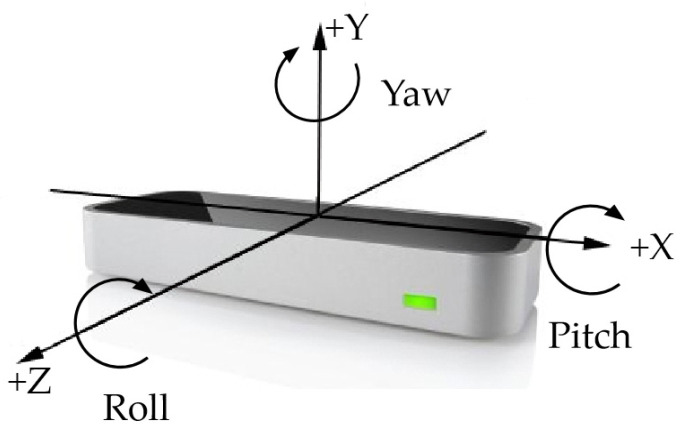
Angle schematic diagram.

This paper takes the Leap Motion body sensor produced by Leap Motion Company (in Figure 1). The Leap Motion device consists of two infrared cameras and multiple LEDs. Its functioning principle relies on capturing hand images illuminated by infrared LEDs through the two cameras, which then analyze gesture changes in the stereo camera images to create a three-dimensional model of the hand. When a hand enters the device’s field of view, the stereoscopic camera system starts capturing the target and calculating the true parallax, thus obtaining spatial information. The sensor’s field of vision is 160° × 160°, allowing for the accurate tracking of hand and finger movements. Its tracking depth ranges from 40 cm to 110 cm, which enhances the range of gesture recognition. Additionally, the camera supports a frame rate of up to 115 frames per second, enabling the quick capture of fast hand movements [22].

**Figure 2:**
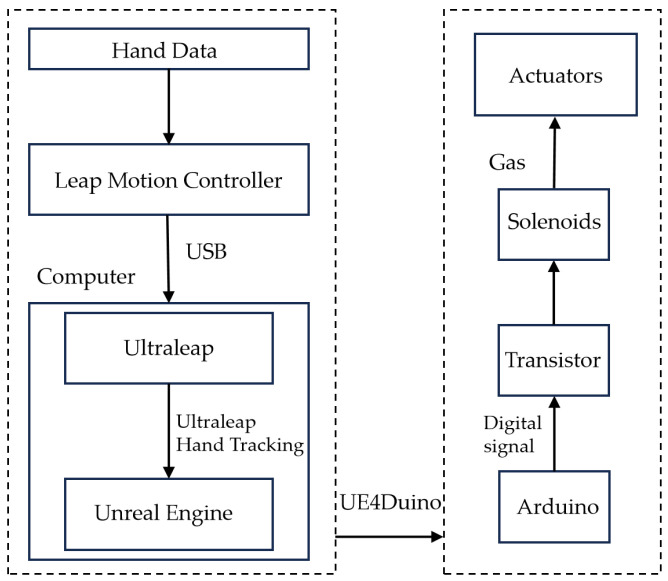
Overall system schematic diagram.

The Leap Motion controller is connected to the computer via USB and accesses hand data through Ultraleap Hand Tracking. To process the acquired hand data effectively, Ultraleap Hand Tracking needs to be installed in Unreal Engine. This setup enables communication between the Leap Motion controller and computing. In Unreal Engine, a gesture recognition blueprint is created to identify specific gestures and store their semantic representations. Communication between Unreal Engine and Arduino is established using UE4Duino, with both systems configured to use the same serial port name and baud rate for easy string-based information transfer. In Arduino, various gesture semantics are associated with corresponding program loops, effectively coordinating gestures with robotic movements. The overall system schematic diagram is shown in Figure 2.

In this study, a Leap Motion sensor device is used to capture the motion of hands and fingers, which records the position, bone structure, and joint angle frame by frame, and stores various data points. To access and manipulate these data using an Unreal Engine, we follow specific procedures: Initially, a data structure is defined in the illusory engine as the basis for creating data tables. The table, structured according to the defined struct, includes columns named after the gesture features to be analyzed. At the same time, the editor blueprint is built to integrate this data table into the illusory engine as an asset. The blueprint takes the raw data from the Leap Motion sensor, converts the data from floating-point format to string format, and processes the data through a series of operations (using the “for each loop” and “append” functions) to continuously populate the data table. The data can then be exported to a CSV file for further analysis. The key components used in the editor blueprint are as follows:

1. Append node: Concatenates texts into a single string;
2. Make Literal String node: Generates text strings that serve as data table headers;
3. Fill Data Table From CSV String node: Fills the data table with the converted string data.

### 2.2. Static Gesture

In the study of static gestures, the key postural features of the hand include the dynamic changes in finger motion and the bending of each finger. Data extraction blueprints can capture features such as fingertip coordinates, fingertip rotation coordinates, palm center coordinates, and palm normal vectors. When the Leap Motion controller detects a hand within its operating range, the blueprint is activated during the Jump-tracking data event. It uses the “for each loop” function to process the hand data, initially identifying and storing the hand type (left or right) in variables in the blueprint. This study mainly focuses on right-hand gestures and collects data from the right hand corresponding to variables. Four static gestures are defined: open palm, clenched fist, extend index finger, and extend index finger with middle finger at the same time.

To determine whether a finger is bent, the “is extended” function is called: if a finger is bent, the Boolean value returned is 0; otherwise, it is 1. Each gesture corresponds to a specific combination of finger states, with the results for all five fingers assessed using the AND logic operator. If all fingers meet the criteria for a particular gesture, the result is true, confirming the detection of that gesture. For the two static gestures, clenched fist and palm open, gesture recognition does not require calling the Boolean function; instead, it relies on specific events to output the recognition result. The palm gesture is recognized by triggering the “grasp on the hand” event, while the fist gesture is recognized by the “release of the hand” event, achieving a recognition accuracy close to 100%. In contrast, using Boolean values from the “is extended” function for the other two gestures yields poor recognition results, leading to potential misrecognition. This issue arises because the “is extended” function does not accurately assess the degree of finger bending.

In order to solve this problem, it is necessary to extract the gesture data at a deeper level. For the gesture with the index finger protruding, the Leap Motion controller obtains the right hand information, then the right index finger information, and then the distal information of the index finger, that is, the fingertip information. Finally, the information on the fingertip rotation vector is obtained. Because the spatial angle of the finger is different, the rotation vector information of the fingertip is also different, so different gestures can be recognized by matching the rotation vector information. The rotation vector information is stored in each frame of data in the form of an array. It contains three pieces of information, which are Pitch value (X value), Yaw value (Y value), and Roll value (Z value), as shown in Figure 1.

By using the Leap Motion controller to invoke the “Print String” command in the blueprint, the coordinates of the fingertips can be obtained when the index and middle fingers are extended. This method allows for printing three angle values in the window. By collecting 50 sets of data, each containing three angle values, the average of each angle across the 50 sets can be calculated. A threshold of ± 5° can then be set based on the average values to achieve a more accurate recognition of specific gestures. The threshold settings for fingertip extension are shown in Table 1

### 2.3. Dynamic Gesture

**Figure 3:**
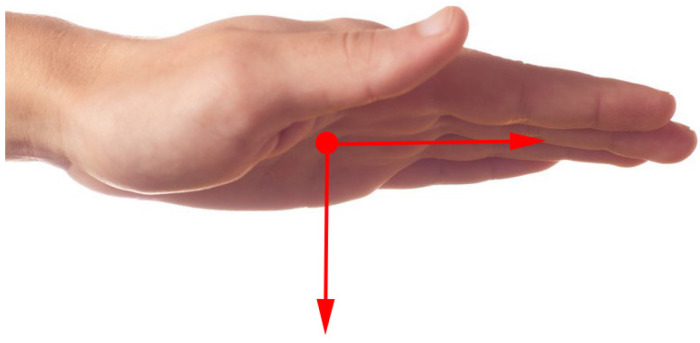
Dynamic gesture features related to palms.

For dynamic gestures, key hand features include palm coordinates, palm velocity, orientation, and the normal direction of the palm. As shown in Figure 3, the palm coordinates are denoted by a red dot at the center of the palm, palm velocity by the speed at which the palm center moves, orientation by the vector from the palm center towards the fingers, and the palm’s normal direction is perpendicular to the orientation of the palm facing inward. Additionally, dynamic gesture characteristics incorporate the center and radius of a sphere, primarily assessing the hand’s degree of curvature, with both the sphere’s center and radius altering as the palm bends.

Our research defines four dynamic gestures: downward palm press, upward palm lift, leftward palm swing, and rightward palm swing. These gestures primarily focus on changes in palm direction rather than the degree of finger bending or palm closure. Consequently, palm velocity and the palm’s normal direction have been identified as key features for dynamic gesture recognition.

Unlike static, dynamic gestures are more prone to disruptions during the collection and recognition phases, such as slight hand tremors that significantly alter the palm’s normal direction. Employing a threshold-based model akin to that used for static gestures—if thresholds are too low, minor hand tremors can cause the Leap Motion controller’s extracted features to exceed these thresholds, preventing the recognition of specific gestures. Conversely, if thresholds are too high, various dynamic gestures may be erroneously recognized as a single gesture. To address this, neural networks can be employed to deeply learn from extensive dynamic gesture data.

Utilizing a blueprint for gesture data extraction, the palm velocities and normal directions for the four gestures are extracted, with each gesture tested thrice. Each test generates a data set containing 200 data sets, with each data set comprising six variables: the X, Y, and Z components of palm velocity and the X, Y, and Z components of the palm’s normal direction, amounting to a total of 2400 data sets.

**Figure 4:**
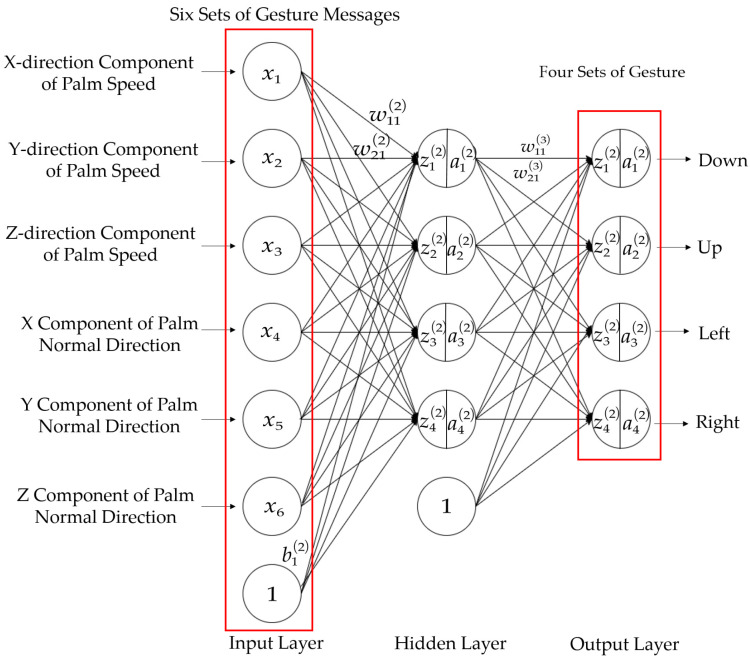
BP neural network diagram.

**Figure 5:**
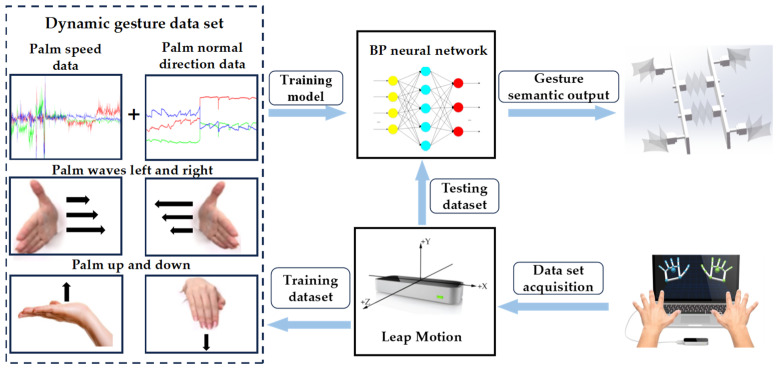
Dynamic gesture recognition process.

The first 2000 sets of data were used as the test set to construct a BP neural network (as shown in Figure 4) with 6 sets of dynamic gesture data as the input layer and 4 dynamic gesture types as the output layer. Dynamic gesture recognition process can be illustrated in Figure 5 $w_{jk}^{\left[l\right]}$ denotes the weight of the connection from the $k^{th}$ neuron in $(l-1{)}^{th}$ of the network pointing to the $j^{th}$ neuron in $l^{th}$. For example, $w_{12}^{\left[1\right]}$ denotes the weight of the connection from the 1st neuron in layer 0 pointing to the 2nd neuron in layer 1. Similarly, we use $b_{j}^{\left[l\right]}$ to denote the deviation of the $j^{th}$ neuron in layer $l^{th}$, use $a_{j}^{\left[l\right]}$ to denote the linear result of the $j^{th}$ neuron in layer $l^{th}$, and use $z_{j}^{\left[l\right]}$ to denote the output of the activation function of the $j^{th}$ neuron in layer $l^{th}$. Since dynamic gesture recognition is a multi-classification problem, the sigmoid function is used as the activation function of the output layer and the Tanh function is used as the hidden layer activation function.

The activation function is denoted by the symbol *σ*, so the activation of the neuron in the $j^{th}$ layer is as follows:

(1) $$a_{j}^{\left[l\right]}=\sigma \left(\sum_{k}w_{jk}^{\left[l\right]}a_{k}^{\left[l-1\right]}+b_{j}^{\left[l\right]}\right),$$

If you use the matrix form to represent the weight matrix, each row is the weight of the lth layer of the connection, for example

(2) $$w^{\left[1\right]}=\left[\begin{array}{cccc} w_{11}^{\left[1\right]} & w_{12}^{\left[1\right]} & \cdots & w_{16}^{\left[1\right]}\\ w_{21}^{\left[1\right]} & w_{22}^{\left[1\right]} & \cdots & w_{26}^{\left[1\right]}\\ w_{31}^{\left[1\right]} & w_{32}^{\left[1\right]} & \cdots & w_{36}^{\left[1\right]}\\ w_{41}^{\left[1\right]} & w_{42}^{\left[1\right]} & \cdots & w_{46}^{\left[1\right]} \end{array}\right],$$

(3) $$b^{\left[1\right]}=\left[\begin{array}{c} b_{1}^{\left[1\right]}\\ b_{2}^{\left[1\right]}\\ b_{3}^{\left[1\right]}\\ b_{4}^{\left[1\right]} \end{array}\right],$$

(4) $$z^{\left[1\right]}=w^{\left[1\right]}a^{\left[0\right]}+b^{\left[1\right]},$$

(5) $$a^{\left[1\right]}=\sigma \left(z^{\left[1\right]}\right),$$

So, for this BP neural network, the forward transfer process is represented by a matrix as follows:

(6) $$C=-\frac{1}{n}\sum_{x}\left[y\ln a^{\left[2\right]}+\left(1-y\right)\ln\left(1-a^{\left[2\right]}\right)\right],$$

In order to find the minimum value of the cost function, backpropagation using gradient descent is required, which constantly changes the weights *w* and biases *b* in the network, so it is necessary to take the partial derivative of *w* and *b*.

The partial derivative of the cost function *C* with respect to $w_{jk}^{\left[l\right]}$ is

(7) $$\begin{array}{ll} \frac{\partial C}{\partial w_{jk}^{\left[l\right]}} & =-\frac{1}{n}\sum_{x}\left[\frac{y_{j}^{\left[l\right]}}{\sigma \left(z_{j}^{\left[l\right]}\right)}-\frac{\left(1-y_{j}^{\left[l\right]}\right)}{1-\sigma \left(z_{j}^{\left[l\right]}\right)}\right]\frac{\partial \sigma \left(z_{j}^{\left[l\right]}\right)}{\partial w_{jk}^{\left[l\right]}}\\ & =-\frac{1}{n}\sum_{x}\left[\frac{y_{j}^{\left[l\right]}}{\sigma \left(z_{j}^{\left[l\right]}\right)}-\frac{\left(1-y_{j}^{\left[l\right]}\right)}{1-\sigma \left(z_{j}^{\left[l\right]}\right)}\right]\sigma^{\prime }\left(z_{j}^{\left[l\right]}\right)a_{j}^{\left[l-1\right]}\\ & =\frac{1}{n}\sum_{x}\frac{\sigma^{\prime }\left(z_{j}^{\left[l\right]}\right)a_{j}^{\left[l-1\right]}}{\sigma \left(z_{j}^{\left[l\right]}\right)\left(1-\sigma \left(z_{j}^{\left[l\right]}\right)\right)}\left(\sigma \left(z_{j}^{\left[l\right]}\right)-y_{i}\right) \end{array},$$

Since the activation function chosen for the output layer is a sigmoid function, so

(8) $$\begin{array}{ll} \sigma ‘\left(z\right) & =\left(\frac{1}{1+e^{-z}}\right)’=\frac{e^{-z}}{{\left(1+e^{-z}\right)}^{2}}=\frac{1+e^{-z}-1}{{\left(1+e^{-z}\right)}^{2}}\\ & =\frac{1}{1+e^{-z}}\left(1-\frac{1}{1+e^{-z}}\right)=\sigma \left(z\right)\left(1-\sigma \left(z\right)\right) \end{array},$$

(9) $$\frac{\partial C}{\partial w_{jk}^{\left[l\right]}}=\frac{1}{n}\sum_{x}a_{j}^{\left[l-1\right]}\left(a_{j}^{\left[l\right]}-y_{i}\right),$$

The partial derivative of the cost function *C* with respect to $b_{j}^{\left[l\right]}$ is

(10) $$\frac{\partial C}{\partial b_{j}^{\left[l\right]}}=\frac{1}{n}\sum_{x}a_{j}^{\left[l\right]}\left(a_{j}^{\left[l\right]}-y_{i}\right),$$

The direction of the gradient descent is already derived from the partial derivatives, after which the step size of the gradient descent, also known as the learning rate, needs to be set, which is given artificially and set to *α*. Finally, the parameters are updated in the opposite direction of the gradient, so

(11) $$b_{j}^{\left[l\right]\prime }=b_{j}^{\left[l\right]}-\alpha \frac{\partial C}{\partial b_{j}^{\left[l\right]}},$$

(12) $$w_{jk}^{{\left[l\right]}^{\prime }}=w_{jk}^{\left[l\right]}-\alpha \frac{\partial C}{\partial w_{jk}^{\left[l\right]}},$$

**Figure 6:**
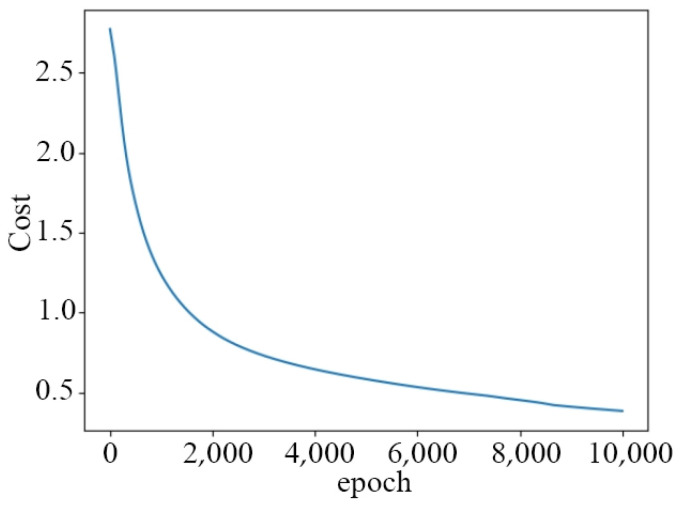
The change diagram of the cost function.

As shown in Figure 6, “epoch” represents the number of iterations and “Cost” represents the value of the cost function. As the number of iterations increases, the cost function value decreases, signifying that the predicted values are getting closer to the actual values. When the iterations of the neural network reach 10,000, the prediction accuracy of the new gesture data is 97.62%.

## 3. Design and Control of Soft Robot

### 3.1. Design

The materials used in traditional soft robots typically include silicone and actuator fabric. These materials possess a certain degree of softness and elasticity, making them suitable for simple movements and morphological changes. In this paper, we propose a novel type of soft crawling robot that utilizes thermoplastic polyurethane (TPU) film for its moving parts. TPU is a material recognized for its excellent elasticity and plasticity. Compared to traditional silicone or actuator fabric, TPU film is lighter and thinner, and more cost-effective. It can achieve a high degree of deformation and distortion, making it well suited for mimicking the movement of biological organisms. The TPU thin film soft crawling robot typically comprises multiple layers of thin films, which facilitate movement through inflation and negative pressure pumping. The elongation of the actuator, which determines the speed of the soft robot, can be controlled by adjusting the number of layers. Additionally, the rotation of the actuator can be adjusted by manipulating the position of the heat seals on each layer, while the spiral motion can be controlled by the offset between the films. This design endows the TPU soft robot with significant flexibility in terms of both adaptability and shape change, presenting promising applications in various fields.

The TPU soft robot is composed of three main parts: an expandable actuator, a curved actuator, and a support plate. Both the expandable actuator and the curved actuator are made of TPU film. The three cavities of the expandable actuator can reach 2 cm after being inflated. The rotation angle of the three cavities of the rotating actuator can reach close to 90° after being inflated.

**Figure 7:**
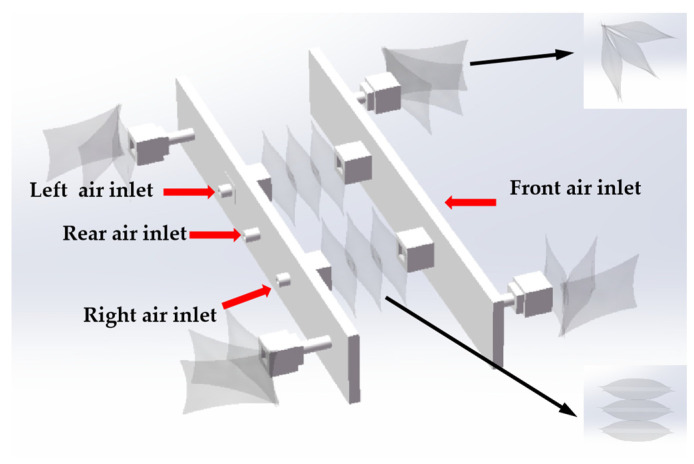
Structure diagram of TPU soft crawling robot.

The support plate is made from 4600 resin and is produced using 3D printing technology. As shown in Figure 7, the interior of the support plate is designed as a cavity to reduce the overall weight of the robotic system and to accommodate the air pathway. At the front end, there is a central air inlet connected to this air pathway, allowing gas to enter the internal cavity. The gas then flows into the quadruped through the air outlets located on both the left and right sides. This process causes the thin film actuator, which forms the quadruped, to bend and create friction with the ground. Additionally, the rear support plate features air inlets on both sides to inflate the left and right expandable actuators.

### 3.2. Control

The soft robot utilizes an Arduino control board as its main control unit. Gesture semantic recognition is achieved by establishing a blueprint in the illusory engine. To effectively match gesture semantics with robot motion, communication between the illusory engine and the Arduino must be established. In the illusory engine, when the clenched fist gesture is recognized, the system prints the string “clenched fist”. In the Arduino program, when the serial port receives the “fist” string, the corresponding command is executed. In essence, by recognizing different gestures and printing different strings in the illusory engine, Arduino receives different strings and executes different loops. Each loop corresponds to a distinct motion state of the soft robot. This process ultimately aligns gesture semantics with the motion states of the soft robot.

The soft crawling robot uses a solenoid valve as the primary control element to control the inflation and deflation of the actuators. By controlling the on/off of the solenoid valve, it regulates the elongation and shortening of the actuator. The coordinated extension and contraction of different actuators enable the soft robot to perform a variety of movements. Additionally, this paper uses an NPN transistor to control the on/off operation of the electromagnetic valve. The base connection control signal of the NPN transistor is managed by the digital signal port of the Arduino. The collector is connected to the load, and the emitter is connected to the negative electrode of the Arduino. When the digital signal port of Arduino is set to high voltage, the transistor turns on, powering the solenoid valve and activating the actuator it controls. Conversely, when the digital signal port is set to low voltage, the transistor disconnects, causing the solenoid valve to lose power and the actuator to return to its original state.

The positive and negative voltage switching circuit is a critical component designed to facilitate the movement of the soft robot. This circuit operates by alternately applying positive and negative pressure to drive the robot’s motion. In this context, positive pressure and negative pressure represent different operational states. When the TPU thin film actuator of the robot needs to expand or bend, positive pressure is introduced into the circuit to inflate the actuator. Conversely, when the actuator needs to contract, negative pressure is applied to extract the gas from the actuator.

**Figure 8:**
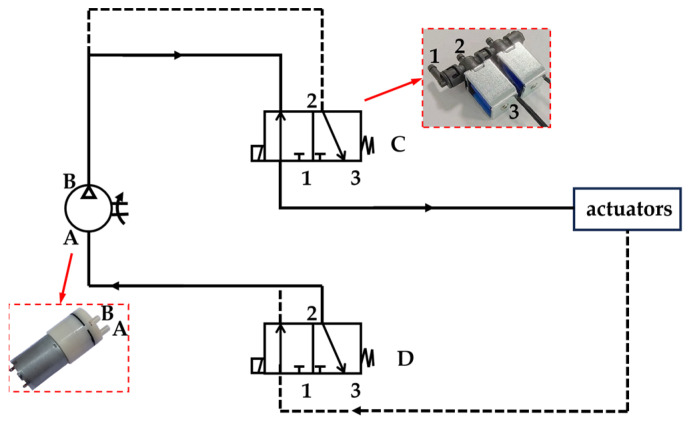
Schematic diagram of positive and negative voltage switching circuits.

The positive and negative pressure switching circuit primarily consists of a negative pressure pump and a two-position, three-way solenoid valve. The specific air pressure circuit is shown in Figure 8. The two solenoid valves depicted are dual valves. Port 1 is an outlet of the solenoid valve, port 2 is an inlet, and port 3 is an outlet. Port 1 serves as the common exhaust port for the two solenoid valves, and upper port 2 is the air inlet. Port 3 at the lower end is designated as the inflatable port. When the solenoid valve is powered, ports 1 and 2 are connected; when the solenoid valve loses power, ports 2 and 3 are connected. The upper end of the pump corresponds to the positive pressure port of port B, and the lower end of the pump connects to the negative pressure port of port A. In the figure, the solid line represents the positive pressure loop, and the dotted line indicates the negative pressure circuit.

The upper part is the C solenoid valve in the dual valve, and the bottom is the D solenoid valve. When the C solenoid valve receives power, and the D solenoid valve loses power, port 1 of the solenoid valve C is connected with port 2, and the air is pumped into the actuator from port B, then the TPU actuator is inflated. At the same time, since port 1 of the D solenoid valve is not connected with port 2, the gas cannot be discharged, so the actuator inflates and expands. When the C solenoid valve loses power and the D solenoid valve is powered, port 1 of the solenoid valve D is connected with port 2, the gas in the actuator is sucked into the A port of the pump, and the TPU actuator is compressed. At the same time, since port 1 of the C solenoid valve is not connected with port 2, the positive pressure circuit will not affect the negative pressure circuit. Through the multiple switching between the positive and negative pressure loops, the length change in the TPU actuator can be achieved to the maximum, and then the movement speed of the soft crawling robot can be improved.

To enable the soft robot to move in a straight line and make turns, it requires at least two expandable actuators. The inlet and outlet of the air must be controlled by solenoid valves, respectively. When the left actuator is inflated by positive pressure and the right actuator is not ventilated or draws negative pressure, the soft robot turns to the right. Conversely, when the right actuator is inflated, and the left actuator is not ventilated or draws negative pressure, the soft robot turns to the left. So, the expandable actuators need two negative pressure pumps and four solenoid valves to control. Additionally, to ensure the stability of the robot, it is equipped with four feet, each ending in a curved actuator. Since the front and rear feet of the robot bend or contract simultaneously, the air path is integrated into the front and rear support plates. This design enables the first two feet to operate using just one pump and two solenoid valves, while the latter two feet require only one pump and two solenoid valves. The whole robot needs eight solenoid valves, four pumps for control, and eight transistors to operate the solenoid valves for turning them on and off.

**Figure 9:**
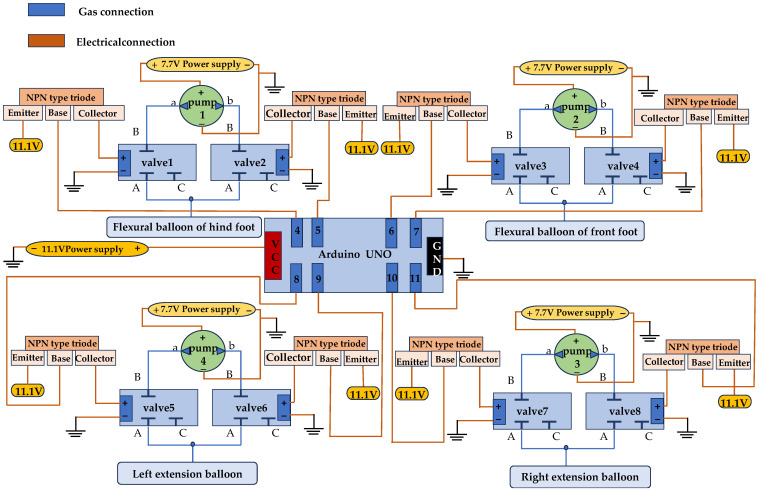
System control.

Firstly, the matching relationship between the Arduino digital ports and solenoid valves is defined, as shown in Table 2. Similarly, we establish the relationships between the pumps, valves, and TPU actuators, as shown in Table 3. After defining the corresponding relationship between the pneumatic components and circuit components, the mechanical and electrical integrated control diagram shown in Figure 9 can be created. In the picture, the orange line represents the circuit connections, and the blue line represents the air connections.

The magnitude of the voltage has a direct impact on the pump’s blade speed, which in turn affects the flow rate of the pump. To prevent the actuator from breaking, the voltage of the pump must not exceed a certain limit; therefore, it is powered by a separate 7.7 V power supply. The digital port of the Arduino is connected to the base of the transistor, and the valve is connected to the transistor through the collector. Both the emitter of the transistor and Arduino are powered by an 11.1 V power supply. Finally, the negative electrodes of both 7.7 V and 11.1 V power supplies are connected to the ground end of the Arduino.

**Figure 10:**
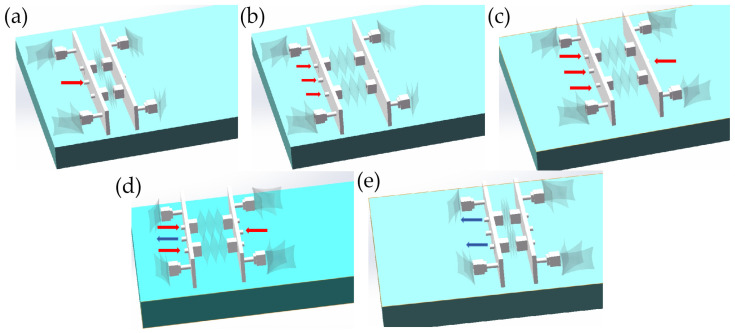
Schematic diagrams of positive and negative voltage switching circuits. (**a**) The curved actuators in the rear are bent. (**b**) The inflatable actuators of the left and right expandable actuators are inflated and expanded. (**c**) The inflatable actuators of the front are inflated and bent. (**d**) The curved actuators in the rear are deflated. (**e**) The actuators of the left and right expandable are deflated.

In the initial state of the soft robot, all the actuators are deflated. When the Arduino port 4 is activated to a high level, valve 1 receives electricity, inflating the two curved actuators in the rear through pump 1. As shown in Figure 10a, the red arrow represents the flow of high-pressure gas, which is pumped into the rear through the central air inlet of the rear support plate. This causes the rear foot to bend backward, creating friction with the ground. This action sets the stage for the next step which involves elongating the actuator so that it can stretch forward.

Due to the sufficient friction between the curved actuator of the rear foot and the ground, the next step is to set the digital ports 8 and 10 of the Arduino to a high level. This action electrifies solenoid valves 5 and 7, allowing high-pressure gas to be pumped into the left and right expandable actuators through pumps 3 and 4, respectively. Consequently, the robot’s overall length increases, as shown in Figure 10b. To enable the robot to move forward as a whole, the front end must maintain friction with the ground. When digital port 6 of the Arduino is set to a high level, the solenoid valve 3 is activated, and high-pressure gas is pumped into the front foot through pump 2. This causes the front foot to bend, generating friction with the ground, as shown in Figure 10c. After these initial steps, both the front foot and rear foot of the robot create friction with the ground. To facilitate forward movement, it is necessary for the actuator of the rear foot to contract, eliminating friction between the actuator and the ground. At this time, it is necessary to set Arduino digital port 4 to a low level and 5 to a high level so that solenoid valve 2 is activated, and the gas in the rear foot is sucked into the pump through pump 1. As shown in Figure 10d, the blue arrow represents negative pressure. Because the forefoot of the robot has friction with the ground, extracting the negative pressure from the two expandable actuators enables the overall forward movement of the robot. At this time, digital ports 8 and 10 of Arduino are set to a low level, digital ports 9 and 11 are set to a high level, solenoid valves 6 and 8 are activated, and the gas in the two expandable actuators is extracted by pumps 3 and 4, resulting in the compression of the two expandable actuators, as shown in Figure 10e.

After completing the initial processes, the robot has successfully moved forward. The final step involves extracting the negative pressure from the front foot actuator before returning to the initial state. Subsequently, the robot will continue executing this cycle as programmed, allowing it to maintain forward movement.

## 4. Motion Experimental Verification of Soft Robot Based on Gesture Recognition

### 4.1. Linear Motion Experiment

**Figure 11:**
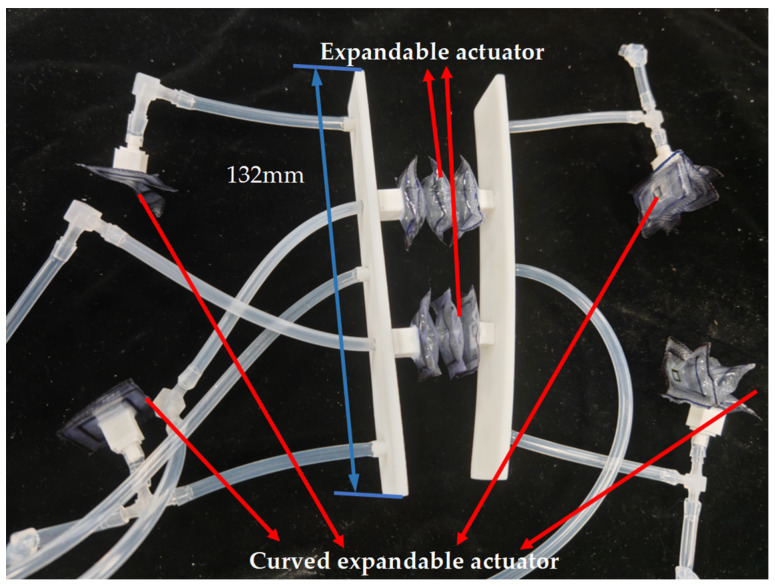
Model of the soft robot.

As shown in Figure 11, this is a model of the soft robot. To achieve the motion of the soft robot, as shown in Table 4, firstly, the matching between the static gesture semantics and the motion of the soft robot is defined. The robot begins to move when it recognizes the fist gesture and stops when it detects the open palm gesture. The relationship between the Arduino digital port and the solenoid valve, as well as the relationship between the pump, valve, and TPU actuator, have been established previously. When Arduino receives the fist gesture message from the illusory engine, it starts executing the command for straight-line motion.

**Figure 12:**
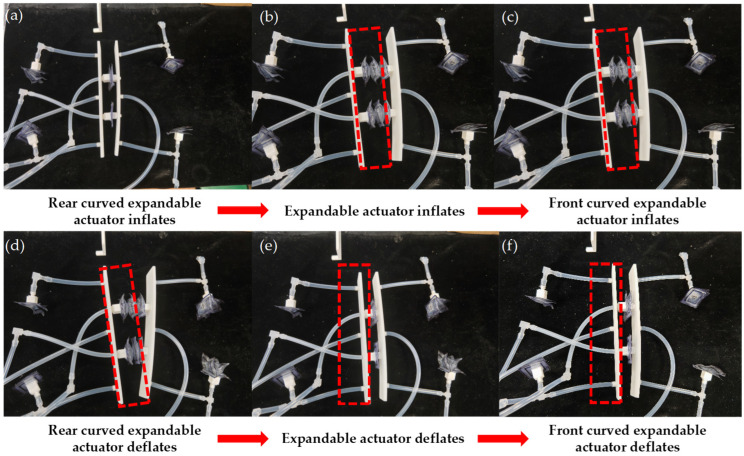
The linear motion of the TPU soft crawling robot (the red dotted line represents the initial position). (**a**) The curved actuators in the rear are bent. (**b**) The inflatable actuators of the left and right expandable actuators are inflated and expanded. (**c**) The inflatable actuators of the front are inflated and bent. (**d**) The curved actuators in the rear are detached from the ground. (**e**) The actuators of the left and right expandable are deflated under negative pressure. (**f**) The curved actuators of the front are detached from the ground.

The specific execution steps are as follows:

(1) The digital port 4 of the Arduino is set to a high level, and the solenoid valve 1 is activated. The two curved expandable actuators in the rear are inflated and bent, resulting in static friction with the ground, as shown in Figure 12a.
(2) The digital ports 8 and 10 of the Arduino are set to a high level, and the solenoid valves 5 and 7 are activated. The left and right expandable actuators are inflated and the robot’s front support plate is forward, as shown in Figure 12b.
(3) The digital port 6 of the Arduino is set to a high level, and the solenoid valve 3 is activated. The actuators of the front are inflated and bent, resulting in friction with the ground, as shown in Figure 12c.
(4) The digital port 4 of the Arduino is set to a low level, the digital port 5 is set to a high level, and the solenoid valve 2 is activated. The two curved expandable actuators in the rear are deflated under negative pressure and detached from the ground, as shown in Figure 12d.
(5) The digital ports 8 and 10 of Arduino are set to a low level, digital ports 9 and 11 are set to a high level, solenoid valves 6 and 8 are activated, two expandable actuators are deflated to negative pressure, and the robot moves forward due to friction between the curved actuators of the front and the ground, as shown in Figure 12e.
(6) The digital port 6 of the Arduino is set to a low level, 7 to a high level, the solenoid valve 4 is activated, and the two front curved actuators are deflated under negative pressure and detached from the ground, as shown in Figure 12f.

### 4.2. Directional Change in Motion Experiment

**Figure 13:**
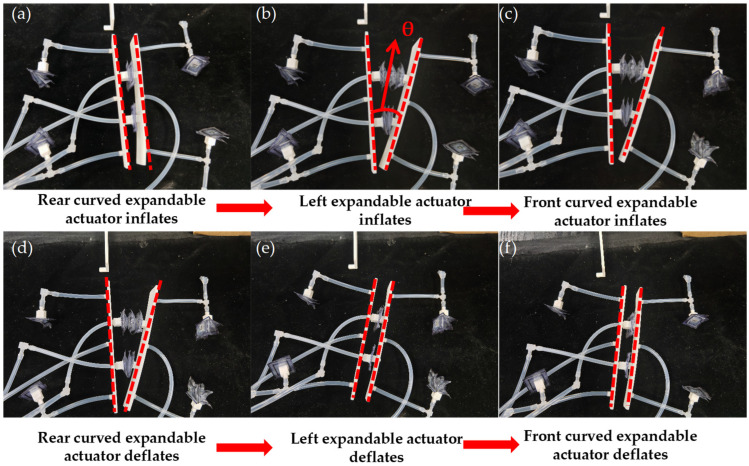
The TPU soft crawling robot changes direction during movement (the red dotted line represents the state of the two plates during the robot’s movement). (**a**) The curved actuators in the rear are bent. (**b**) The inflatable actuators of the left expandable actuators are inflated and expanded. (**c**) The inflatable actuators of the front are inflated and bent. (**d**) The curved actuators in the rear are detached from the ground. (**e**) The actuators of the left expandable are deflated under negative pressure. (**f**) The curved actuators of the front are detached from the ground.

Firstly, the matching relationship between the semantics of static gesture and the motion of the soft robot is defined, and the index finger is extended as the right turn of the robot, and the index finger and middle finger are extended as the left turn of the robot at the same time. The definition of motion and stop gesture is the same as 4.1. The matching relationship between the static gestures and robot motion is shown in Table 4. When Arduino receives the gesture semantics of the index finger sticking out from the illusory engine, it starts to execute the robot right turn command. The specific execution process is as follows:

(1) The digital port 4 of the Arduino is set to a high level, the solenoid valve 1 is activated, and the two curved expandable actuators in the rear are inflated and bent, resulting in static friction with the ground, as shown in Figure 13a;
(2) The digital port 8 of the Arduino is set to a high level, the solenoid valve 5 is activated, and the left expandable actuator is inflated to make the front support plate of the robot rotate at a certain angle, as shown in Figure 13b;
(3) The digital port 6 of the Arduino is set to a high level, and the solenoid valve 3 is activated, and the two front curved expandable actuators are inflated and bent, resulting in friction with the ground, as shown in Figure 13c;
(4) The digital port 4 of the Arduino is set to a low level, the digital port 5 is set to a high level, the solenoid valve 2 is powered, and the two curved expandable actuators in the rear are deflated under negative pressure and detached from the ground, as shown in Figure 13d;
(5) Arduino’s digital port 8 is set to a low level, digital port 9 is set to a high level, the solenoid valve is activated, the left expandable actuator is deflated to negative pressure, and the robot completes steering, as shown in Figure 13e;
(6) The digital port 6 of the Arduino is set to a low level, 7 to a high level, the solenoid valve 4 is activated, and the two front curved expandable actuators are deflated under negative pressure and detached from the ground, as shown in Figure 13f.

Whenever the six steps described are executed, the robot rotates a specific angle to the right. If a larger angle is required, the robot can simply repeat this cycle while keeping the forefinger extended. It will continue to rotate to the right until it reaches the desired angle. After turning, the robot will move in a straight line, stopping only when the fist is clenched. The process for turning left is similar. When the Arduino detects that both the index finger and middle finger are extended simultaneously, it initiates the command for turning left. Unlike the right turn, the right actuator is inflated and extended during step (2).

To summarize the overall flow of the robot’s movement, when the device is powered on and the fist is clenched, the robot begins to move. To turn right, extend the index finger and rotate to the specified angle; then return to the clenched fist gesture to move in a straight line. For a left turn, extend both the index and middle fingers at the same time, rotate to the desired angle, and then switch back to the fist gesture to continue moving straight. To command the robot to stand still, simply open your palms.

## 5. Conclusions

In this paper, a gesture recognition-based method was proposed to interact with and control a TPU-based soft crawling robot. The system used Leap Motion sensors to collect gesture data and implemented static and dynamic gesture recognition models using a threshold-based approach and a BP neural network, respectively. A novel soft robot design using TPU films was introduced to reduce cost and increase adaptability. In addition, a fully integrated electromechanical control system was developed using transistor switching circuits and a positive/negative pressure switching circuit to optimize the robot’s motion.

The experimental results showed that the soft crawling robot could successfully perform linear and directional movements under static gesture control, validating the effectiveness of the proposed methods. The design also demonstrated the advantages of low cost, easy fabrication, and high adaptability. Future work will focus on improving the robustness of dynamic gesture recognition and extending the functionality of the robot to more complex movements and applications.

## Figures and Tables

**Table 1:** Threshold values corresponding to the extended fingertip.

Tip of Finger	Threshold Value
Index finger	X value: −115 ± 5°; Y: 50 ± 5°; Z: 71 ± 5°
Middle finger	X value: −125 ± 5°; Y: 63 ± 5°; Z: 62 ± 5°

**Table 2:** Arduino digital port and solenoid valve matching relationship table.

Arduino Digital Port	4	5	6	7	8	9	10	11
Solenoid valve number	1	2	3	4	5	6	7	8

**Table 3:** Matching relation table of pump, valve, and TPU actuator.

Actuator	Pump	Valve
Two curved actuators in the rear	1	1, 2
Two curved actuators in the front	2	3, 4
Left expandable actuator	3	5, 6
Right expandable actuator	4	7, 8

**Table 4:** Static gesture and robot rectilinear motion matching table.

Gesture	Robot Motion
Make a fist	Start moving
Open palms	Stop moving
Extend the index finger	Turn right
Extend the index finger and middle finger at the same time	Turn left

## Data Availability

Data are contained within the article.
